# Geographic social inequalities in information-seeking response to the COVID-19 pandemic in China: longitudinal analysis of Baidu Index

**DOI:** 10.1038/s41598-022-16133-2

**Published:** 2022-07-18

**Authors:** Zhicheng Wang, Hong Xiao, Leesa Lin, Kun Tang, Joseph M. Unger

**Affiliations:** 1 Vanke School of Public Health, Tsinghua University, No 30 Shuangqing Road, Beijing, 100084 China; 2 School of Medicine, Tsinghua University, Beijing, China; 3 China Development Research Foundation, Beijing, China; 4 Public Health Sciences Division, Fred Hutchinson Cancer Research Center, Seattle, WA 98109 USA; 5 Department of Infectious Disease Epidemiology, London School of Hygiene and Tropical Medicine, London, UK; 6 Laboratory of Data Discovery for Health (D24H), Hong Kong Science Park, Sha Tin, Hong Kong Special Administrative Region China

**Keywords:** Epidemiology, Psychology and behaviour

## Abstract

The outbreak of the COVID-19 pandemic alarmed the public and initiated the uptake of preventive measures. However, the manner in which the public responded to these announcements, and whether individuals from different provinces responded similarly during the COVID-19 pandemic in China, remains largely unknown. We used an interrupted time-series analysis to examine the change in Baidu Search Index of selected COVID-19 related terms associated with the COVID-19 derived exposure variables. We analyzed the daily search index in Mainland China using segmented log-normal regressions with data from Jan 2017 to Mar 2021. In this longitudinal study of nearly one billion internet users, we found synchronous increases in COVID-19 related searches during the first wave of the COVID-19 pandemic and subsequent local outbreaks, irrespective of the location and severity of each outbreak. The most precipitous increase occurred in the week when most provinces activated their highest level of response to public health emergencies. Search interests increased more as Human Development Index (HDI) -an area level measure of socioeconomic status—increased. Searches on the index began to decline nationwide after the initiation of mass-scale lockdowns, but statistically significant increases continued to occur in conjunction with the report of major sporadic local outbreaks. The intense interest in COVID-19 related information at virtually the same time across different provinces indicates that the Chinese government utilizes multiple channels to keep the public informed of the pandemic. Regional socioeconomic status influenced search patterns.

## Introduction

In late December 2019, a new type of acute respiratory syndrome, which was later known as coronavirus disease—COVID-19, was first reported in Wuhan, China. This unknown coronavirus caused public alarm in Chinese reminiscent of the SARS outbreak in 2003. Subsequently, the massive media coverage surrounding the official confirmation of human-to-human transmission of COVID-19 on 20 January 2020 further alarmed the Chinese public, who then actively sought out related information online^1^. The public’s information-seeking behaviors in general and in particular as it related to COVID-19 can be captured by the use of data from Internet search engines^2^.

Epidemiological research has shown how the collective attention of the public changed regarding COVID-19 in early 2020. Studies have demonstrated that search volumes for COVID-19 related words increased during the first waves of COVID-19, especially the declaration of a Public Health Emergency of International Concern (PHEIC) by World Health Organization on 12 March 2020, but then fell to a much lower levels in April 2020^3–5^. Many countries have taken the "flatten the curve" strategy at early 2020^6^. In such countries, the public writ large may consider COVID-19 case fluctuations to be the norm. In contrast, since the first wave of COVID-19 in early 2020, China has adopted a "zero COVID″ policy, implementing a series of non-pharmaceutical interventions (NPIs), including contact tracing and isolation, strict border control, and massive testing. From 21 Feb to 17 March 2020, the average number of reported daily domestic new COVID-19 cases was under ten, and from 18 Mar to 28 April, there were no additional reported domestic COVID-19 cases^1^. Nevertheless, subsequent waves of COVID-19 did occur due to relaxed quarantines, leading to locally transmitted infections, in part due to imported cases^1^. These subsequent domestic COVID-19 outbreaks, though minor, were nonetheless widely reported by mass media including newspaper, television and online platforms, which raised public alarm. Patterns of information-seeking related to these subsequent outbreaks have yet to be studied.

Previous survey-based studies have shown that individuals with lower socioeconomic status have lower awareness, concern and knowledge about certain infectious disease during the outbreaks^7–10^. Therefore, collective public information-seeking behaviors may vary across the 31 provinces in China in relation to differences in socioeconomic development^11^. However, few studies about inequities in awareness or response to COVID-19 in China have been conducted to date, in part due to the failure to capture the social determinants in the health information system in China. Addressing health inequities against COVID-19 is urgently needed^12^, since such inequities can exacerbate existing social inequalities^13^. Many adolescents and adults are using the internet to diagnose themselves or learn about health concerns^14–17^; in this scenario, internet access and use becomes an increasingly important tool to improve health literacy and potentially health outcomes^18–20^. The number of internet users in China exceeded one billion as of August 2021^21^. Thus, public knowledge, awareness of and concerns about COVID-19 can be measured by search interests in COVID-19 related terms. The investigation of the intensity of internet search interests can therefore be used to examine potential differences in patterns of information-seeking reactions (and by extension, health literacy and health outcomes) regarding the pandemic by levels of socioeconomic status^16,19,22,23^.

## Results

The median of the national-level daily search index for Covid-19 related terms was 4, 533 (IQR (Interquartile Range) = 1, 301) before the COVID-19 outbreak (January 1 2017 to December 30 2019), and 314, 718 (IQR = 445, 074) after the outbreak (December 31 2019 to March 15 2021). The median of the provincial-level search index, ranged from 63 (IQR = 7) in Tibet to 1138 (IQR = 302) in Guangdong before COVOD-19, and ranged from 1386 (IQR = 983) in Tibet to 38, 061(IQR = 45, 784) in Guangdong after the COVID-19 outbreak. The crude relative change in the median of the search index ranged from 2 099% in Tibet and 2 034% in Hainan to 3 872% in Beijing and 4 284% in Liaoning (Table 1). 89, 936 cases of SARS-COV-2 occurred nationwide (ranging from 1 case in Tibet to 68, 021 cases in Hubei) from December 31, 2020 to March 15, 2021. The number of confirmed cases outside Tibet and Hubei ranged from 18 (0.1%) in Qinghai to 2, 245 (10.6%) in Guangdong province. In conjunction with these search patterns, 13%, 76% and 11% of confirmed Covid-19 cases were reported in January 2020, February 2020 and from March 2020 to March 2021 respectively.

**Table 1:** Comparison of search index in the COVID-19 and pre-COVID period.

	Pre-Covid-19 period (Jan 1 2017–Dec 30 2019)		Covid-19 period (Dec 31 2019–Mar 15 2021)		Relative change (%)
	Median	IQR	Median	IQR	
**Low HDI**					
Tibet	63	7	1386	983	2099
Yunnan	290	79	7939	7612	2642
Guizhou	224	87	7011	6531	3030
Gansu	175	74	5304	6348	2931
Qinghai	73	33	2465	1625	3277
Xinjiang	176	56	6108	7744	3370
Guangxi	303	119	7548	9863	2391
Sichuan	536	167	15,792	19,522	2846
Anhui	324	124	11,199	13,205	3356
Ningxia	86	59	2569	2019	2887
**Middle HDI**					
Jiangxi	274	96	8021	8726	2827
Henan	489	152	15,319	17,469	3033
Hebei	435	152	18,986	26,505	4270
Hunan	348	124	11,216	13,615	3123
Shanxi	263	84	8674	11,963	3198
Hainan	157	53	3351	2709	2034
Chongqing	272	97	7965	8692	2828
Heilongjiang	279	88	10,902	15,782	3808
Shaanxi	356	116	9111	11,704	2463
Hubei	404	137	10,723	11,385	2554
Fujian	462	155	10,842	11,205	2247
Inner Mongolia	207	72	6747	8116	3167
Jilin	234	76	9375	12,607	3906
**High HDI**					
Shandong	597	210	21,802	31,866	3555
Guangdong	1138	302	38,061	45,784	3246
Liaoning	365	144	16,001	24,060	4284
Zhejiang	754	250	22,516	26,850	2886
Jiangsu	789	253	23,453	30,053	2874
Tianjin	247	84	7516	8409	2943
Shanghai	622	194	16,430	19,065	2541
Beijing	647	204	25,699	36,265	3872

### Model estimated change of search index by human development index (HDI) categories

#### Pre-Covid-19

**Figure 1:**
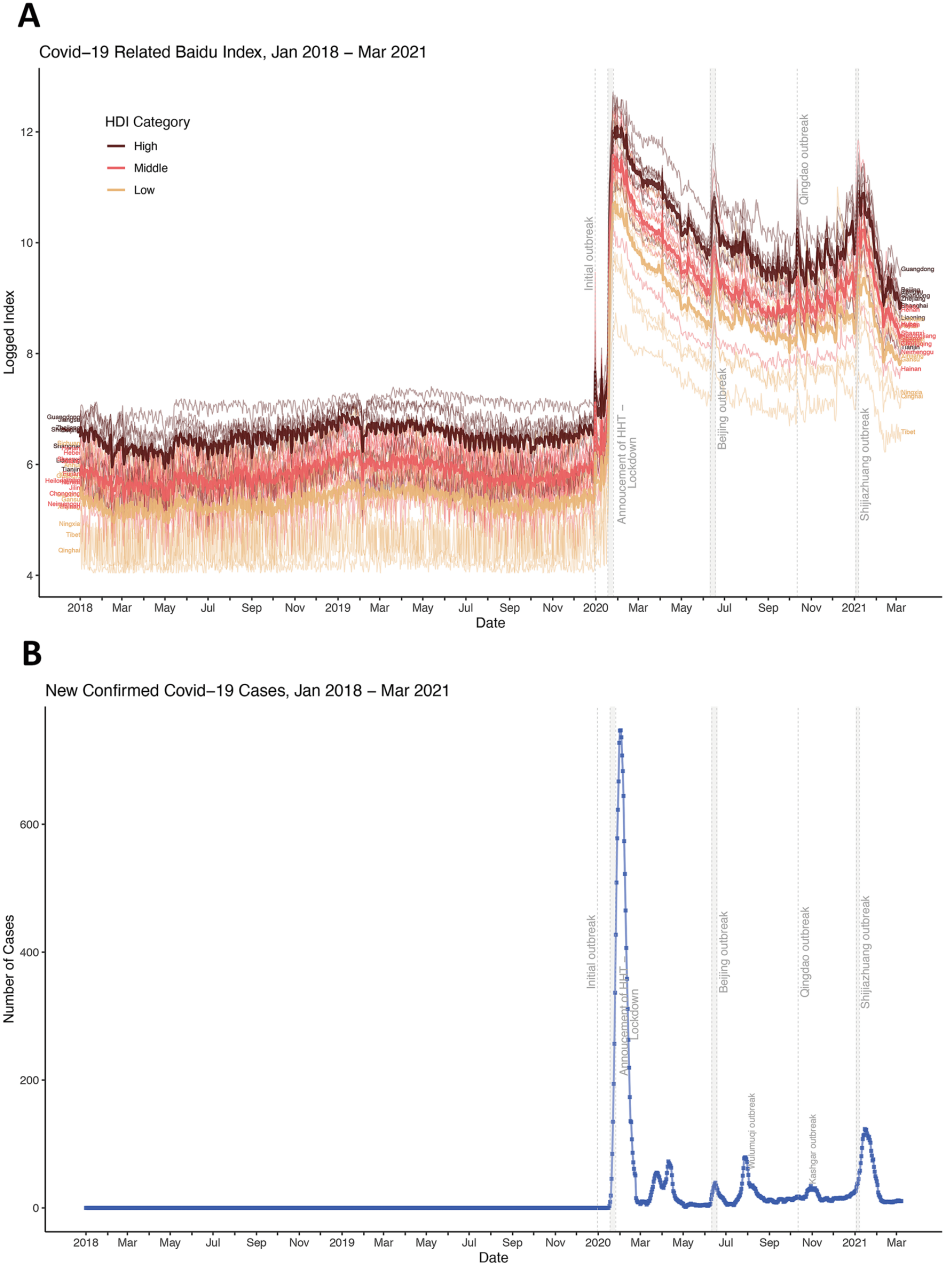
Baidu search index by province and number of new confirmed cases over time. (**A**) Observed daily search index (log transformed) by province and HDI category over time. Aggregated search index by HDI category over time is shown in Fig. S1. (**B**) Daily new confirmed COVID-19 in China (cases in Hubei provinces are excluded).

As shown in Table 2, there was a 10% (relative risk (RR) = 1.10, 95% CI 1.07–1.13, p < 0.0001), 11% (RR = 1.11, 95% CI 1.08–1.14, p < 0.0001) and 13% (RR = 1.13, 95% CI 1.10–1.16, p < 0.0001) annual increase in the search index before the pandemic among regions with low, middle and high HDI respectively. The difference in pre-Covid-19 trends of the search index among the three HDI groups was not statistically significant (middle vs. low, ratio of RR = 1.01, p = 0.6188; high vs. low, ratio of RR = 1.03, p = 0.2239) (Table 2, Fig. 1).

**Table 2:** Model estimated change of search index by HDI categories.

	Regions with Low HDI			Regions with Middle HDI				Regions with High HDI			
	RR (95% CI)	p-value	Ratio of RR	RR (95% CI)	p-value	Ratio of RR*	p-value	RR (95% CI)	p-value	Ratio of RR*	p-value
**Pre-Covid-19**											
Yearly change Jan 1 2016–Dec 30 2019	1.10 (1.07, 1.13)	< 0.0001	Reference	1.11 (1.08, 1.14)	< 0.0001	1.01 (0.97, 1.05)	0.6188	1.13 (1.10, 1.16)	< 0.0001	1.03 (0.98, 1.07)	0.2239
**Initial COVID-19 wave**											
Level change on Dec 31 2019	1.41 (1.34, 1.49)	< 0.0001	Reference	1.62 (1.54, 1.70)	< 0.0001	1.15 (1.07, 1.23)	0.0002	1.58 (1.48, 1.68)	< 0.0001	1.12 (1.03, 1.21)	0.0091
Level change Jan 18 (HHT announced)–Jan 25 2020 (lockdown)	106.80 (100.07, 113.99)	< 0.0001	Reference	124.55 (117.61, 131.90)	< 0.0001	1.16 (1.07, 1.27)	0.0004	125.31 (116.53, 134.75)	< 0.0001	1.17 (1.06, 1.30)	0.0012
Weekly change Jan 25—Jun 10 2020	0.90 (0.89, 0.90)	< 0.0001	Reference	0.89 (0.88, 0.89)	< 0.0001	0.99 (0.98, 0.99)	< 0.0001	0.89 (0.89, 0.90)	< 0.0001	0.99 (0.99, 1.00)	0.0768
**Beijing outbreak**											
Level change Jun 11- Jun 17 2020	1.91 (1.79, 2.03)	< 0.0001	Reference	1.34 (1.26, 1.42)	< 0.0001	1.01 (0.94, 1.10)	0.7419	2.12 (1.98, 2.27)	< 0.0001	1.11 (1.01, 1.21)	0.0227^b^
Weekly change Jun 17—Oct 11 2020	0.96 (0.95, 0.96)	< 0.0001	Reference	1.02 (1.01, 1.02)	< 0.0001	0.99 (0.98, 1.00)	0.0059	0.94 (0.93, 0.94)	< 0.0001	0.98 (0.97, 0.99)	< 0.0001
**Qingdao outbreak**											
Level change on Oct 12th	1.31 (1.23, 1.40)	< 0.0001	Reference	1.34 (1.26, 1.42)	< 0.0001	1.02 (0.93, 1.11)	0.6979	1.41 (1.31, 1.52)	< 0.0001	1.07 (0.97, 1.18)	0.1693
Weekly change in winter wave Oct 12 2020–Jan 3 2021	1.01 (1.00, 1.01)	0.0647	Reference	1.02 (1.01, 1.02)	< 0.0001	1.01 (0.99, 1.02)	0.1043	1.02 (1.01, 1.03)	0.0002	1.01 (0.99, 1.02)	0.1058
**Shijiazhuang outbreak**											
Level change Jan 3–Jan 7 2021	2.00 (1.85, 2.16)	< 0.0001	Reference	2.67 (2.50, 2.86)	< 0.0001	1.34 (1.21, 1.48)	< 0.0001	2..45 (2.24, 2.67)	< 0.0001	1.22 (1.09, 1.37)	0.0007
Weekly change Jan 7–Mar 15 2021	0.83 (0.82, 0.84)	< 0.0001	Reference	0.80 (0.79, 0.80)	< 0.0001	0.95 (0.94, 0.97)	< 0.0001	0.78 (0.77, 0.79)	< 0.0001	0.94 (0.93, 0.96)	< 0.0001

*In reference to the low HDI category.

^b^Not significant, after applying Holm-Bonferroni adjustment to maintain a family-wide type I error rate of 0.05 (All p values for the tests that are not marked ^“b”^ are significant unless they are greater tan 0.05).

#### Initial COVID-19 wave

During the initial wave, the search index increased by 41%, 62% and 58% on December 31, 2019 among regions with low (RR = 1.41, 95% CI 1.34–1.49, p < 0.0001), middle (RR = 1.62, 95% CI 1.54–1.70, p < 0.0001) and high (RR = 1.58, 95% CI 1.48–1.68, p < 0.0001) HDI, respectively. The immediate increase in middle and high HDI regions was statistically significantly higher than the increase in low HDI regions (middle vs. low, ratio of RR = 1.15, p = 0.0002; high vs. low, ratio of RR = 1.12, p = 0.0091).

Similarly, there was a 107-fold, 125-fold and 125-fold increase in search index between January 18 and January 25 2020, the period shortly after the official announcement of human-to-human transmission (HHT), among regions with low (RR = 106.8, 95% CI 100.1–114.0, p < 0.0001), middle (RR = 124.6, 95% CI 117.6–131.9, p < 0.0001) and high (RR = 125.3, 95% CI 116.5–134.8, p < 0.0001) HDI, respectively. The immediate increase in this short period among middle and high HDI regions were statistically significantly higher than the increase in low HDI regions (middle vs. low, ratio of RR = 1.16, p = 0.0004; high vs. low, ratio of RR = 1.17, p = 0.0012). From the peak of the search index on January 25 to June 10 2020, a 10%, 11% and 11% decrease per week was observed in the search index among regions with low (RR = 0.90, 95% CI 0.89–0.90, p < 0.0001), middle (RR = 0.89, 95% CI 0.88–0.89, p < 0.0001) and high (RR = 0.89, 95% CI 0.89–0.90, p < 0.0001) HDI, respectively (Table 2).

#### Beijing outbreak

The outbreak in Beijing was associated with a 91%, 34% and 112% increase in the search index among regions with low (RR = 1.91, 95% CI 1.79–2.03, p < 0.0001), middle (RR = 1.34, 95% CI 1.26–1.42, p < 0.0001) and high (RR = 2.12, 95% CI 1.98–2.27, p < 0.0001) HDI, respectively, in the first week (June 11–17 2020) of the outbreak. Additionally, the Beijing outbreak was associated with an increase in the monthly change rate of the search index. From June 17 to October 11 2020, a 4% decrease, 2% increase and 6% decrease per month in the search index was observed among regions with low (RR = 0.96, 95% CI 0.95–0.96, p < 0.0001), middle (RR = 1.02, 95% CI 1.01–1.02, p < 0.0001) and high (RR = 0.94, 95% CI 0.93–0.94, p < 0.0001) HDI, respectively (Table 2).

#### Qingdao outbreak

The Qingdao outbreak was associated with a comparable 31%, 34% and 41% immediate increase in the search index among regions with low (RR = 1.31, 95% CI 1.23–1.40, p < 0.0001), middle (RR = 1.34, 95% CI 1.26–1.42, p < 0.0001) and high (RR = 1.41, 95% CI 1.31–1.52, p < 0.0001) HDI, respectively. In the winter wave after the Qingdao outbreak, search index increased by 1%, 2% and 2% per week among regions with low (RR = 1.01, 95% CI 1.00–1.01, p = 0.0647), middle (RR = 1.02, 95% CI 1.01–1.02, p < 0.0001) and high (RR = 1.02, 95% CI 1.01–1.03, p = 0.0002) HDI, respectively.

#### Shijiazhuang outbreak

**Figure 2:**
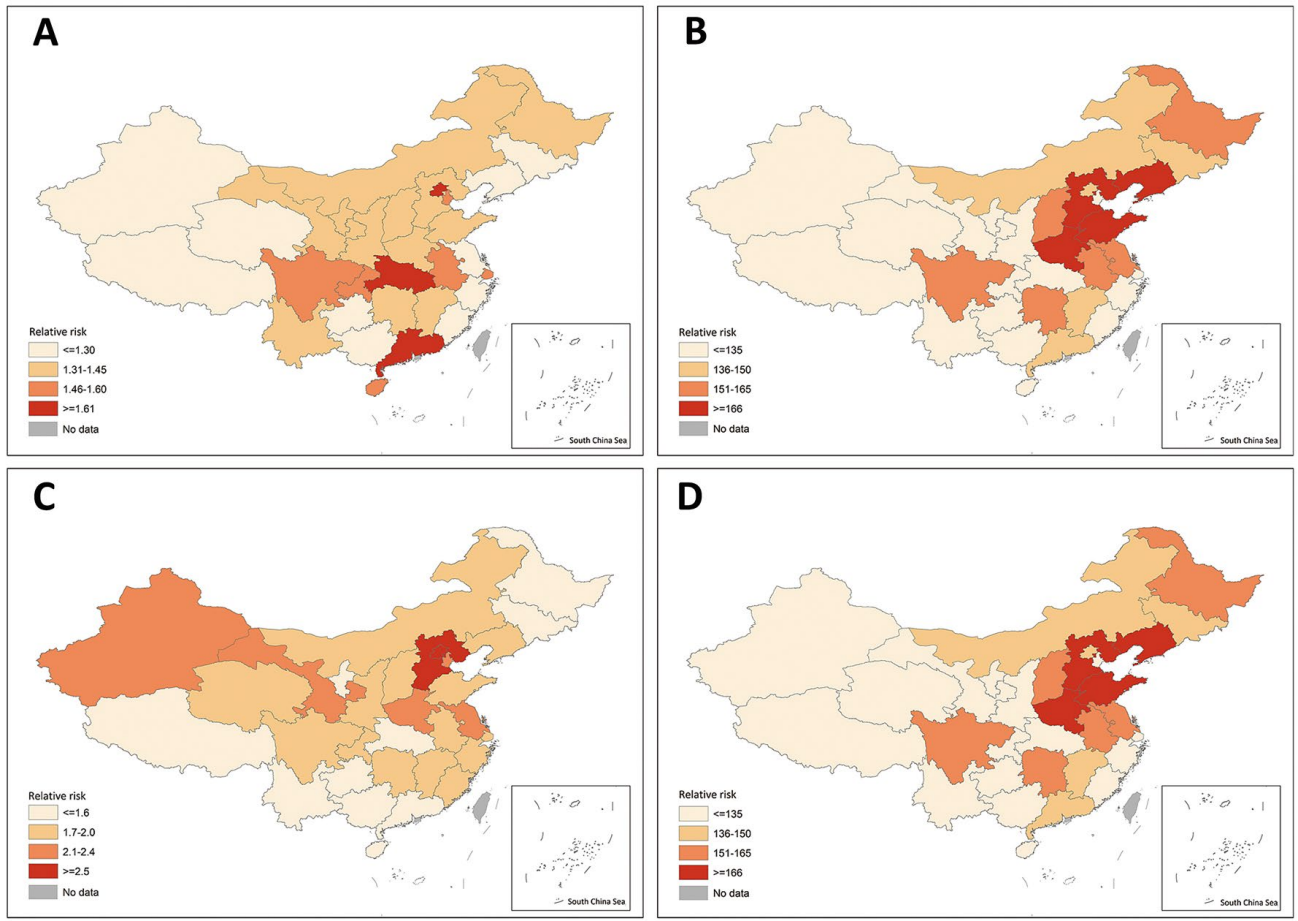
Immediate relative change in search index at different exposure period (**A**) December 31 2019, the estimated start of the first Covid-19 wave. (**B**) 18 January 18 2020 (official announcement of human-to-human transmission) to Jan 25 January 2020 (shortly after the lockdown and the estimated peak of daily search index in the initial Covid-19 wave). (**C**) Outbreak in Beijing starting on June 11 2020. (**D**) Outbreak in Shijiazhuang starting on January 3 2021. Specific point estimate for relative change and the corresponding 95% CIs are provided in the supplemental materials.

The Shijiazhuang outbreak in January 2021 was associated with a 100%, 167% and 145% immediate increase in search index among regions with low (RR = 2.00, 95% CI 1.85–2.16, p < 0.0001), middle (RR = 2.67, 95% CI 2.50–2.86, p < 0.0001) and high (RR = 2.45, 95% CI 2.24–2.67, p < 0.0001) HDI. In regions with low HDI (middle vs. low, ratio of RR = 1.34, p < 0.0001; high vs. low, the ratio of RR = 1.22, p = 0.0007). However, the 20% and 22% weekly decrease in search index after the Shijiazhuang outbreak among regions with middle (RR = 0.80, 95% CI 0.79–0.80, p < 0.0001) and high (RR = 0.78, 95% CI 0.77–0.79, p < 0.0001) HDI, respectively, was statistically significantly greater (p < 0.0001) than the 17% monthly decrease in the region with low HDI (RR = 0.83, 95% CI 0.82–0.84, p < 0.0001). Figure 2 illustrated the heterogeneity in the immediate relative change in the search index following each pre-specified exposure across the country.

### Association between HDI, GNP per person, education, life expectancy and magnitude of change in the search index

The results from models where HDI or its component was coded as a continuous variable were consistent with findings from our main analysis. As shown in Table S1, the pre-pandemic trends in two provinces differing in HDI, GNPPP (Gross national product per person), education year or life expectancy by one standard deviation were similar (p > 0.1). The immediate relative increase in the search index in a province with one standard higher HDI was statistically higher (initial wave: ratio of RR = 1.09, p < 0.0001; HHT announcement: ratio of RR = 1.04 p = 0.0395; Beijing outbreak: ratio of RR = 1.06, p = 0.0090; Qingdao outbreak: ratio of RR = 1.04, p = 0.0324; Shijiazhuang outbreak: ratio of RR = 1.11, p < 0.0001). In contrast, the gradual decrease in the search index in a province with one standard deviation higher HDI after each exposure was either similar or greater. For each exposure, the difference associated with GNPPP, education year or life expectancy in the directions and magnitudes of both immediate and gradual effect across provinces was similar to the difference associated with HDI.

## Discussion

The study used the Baidu search index related to COVID-19 at the subnational level to analyze the search volume of Chinese Internet users for COVID-19, which was used to reflect the level of public awareness of COVID-19, and the differences in levels of awareness of and proactive information-seeking response to COVID-19 in different regions. Our study found that, in January 2020, the outbreak of the Wuhan epidemic triggered an increase in search terms for COVID-19 among Internet users in different regions. In particular, this increasing trend was most sharply observed between January 18–25, 2020, a period when mass media (e.g. television, radio, newspaper and online media) reported the confirmation of human-to-human transmission of SARS-CoV-2, greatly increasing public awareness of the threat of the disease. This was reflected in a huge increase in search indices. In the later outbreaks, we also found that each subsequent outbreak in China reignited public interest in COVID-19, which resulted in the increasing search volume for COVID-19-related keywords. However, the subsequent increase in COVID-19 searches did not surpass the first search index apex, which may be explained by individuals having accumulated prior knowledge already and becoming more accustomed to subsequent COVID-19 outbreaks, as well as by the fact that subsequent outbreaks were less severe.

When the Wuhan municipal government issued a notification about the existence of unknown respiratory syndrome at the end of December 2021, the public response was reminiscent of the fear caused by SARS in 2003, especially as little was known about this new pneumonia. On January 20, the confirmation of human-to-human transmission of COVID-19 was announced via mass media. After being informed of their susceptibility to COVID-19, the public across China rushed to seek related information online^24,25^.These increases happened in just 3 days, from 20 to 23 Jan. In contrast, global collective public attention to COVID-19 reached its peak on 12 March, following the declaration of PHEIC by the World Health Organization^26,27^. The surge of public collective attention to COVID-19 in China during the early stage of the outbreak could be attributed to governments at all levels mobilizing the whole society to contain the COVID-19 in China^28^. In addition, the first spike in search volume for COVID-19 related keywords occurred that same day across all provinces in China, which was different from the subnational patterns in the US where state-level search volume typically peaked at the time the first COVID-19 case was announced in the state^2,3,29,30^.

We further found that, after the first information-seeking peak, although there was an evident decline in the search interests in COVID-19 related words from February to April 2021, the public concern (reflected by the search interests) about the COVID-19 pandemic remained at a high level in every province and through the end of our study period. As Chinese government took the nationwide, stringent non-pharmaceutical interventions, China saw success in its initial containment of COVID-19, as the daily new local cases were under 10 from late March to late April. In the context of the zero-COVID policy in China, and few new cases of COVID-19, news of any new domestically occurring cases of COVID-19 in China generated a relatively large amount of media attention. For example, in Beijing in June 2020, a sporadic outbreak of 335 new COVID-19 cases (and no deaths) occurred due to imported frozen products^31^. Despite the limited nature of this local outbreak, it nonetheless generated intense public interest in Beijing and throughout the country. Such increased attention to new COVID-19 outbreaks in China suggests widespread, enduring awareness among the public about the ongoing risks of COVID-19.

We found social inequalities in information-seeking behavior intensity within China. Studies have confirmed that deprived populations show relatively lower awareness of infectious diseases, including H1N1 and COVID-19^10,32,33^. In our findings, these inequalities are evidenced by the absolute change in Baidu Index volume as well as the speed with which peak search volume is achieved. Populations in areas with higher human development showed a higher volume of COVID-19 related searches, and their searches increased faster and maintained a relatively lower decline rate, which suggests not only the population living in HDIs areas have a faster response to COVID-19 but also maintained a heightened, more durable awareness about the COVID-19 epidemic. Lower awareness of COVID-19 may result in less attention paid to personal mitigation techniques and lower compliance with non-pharmaceutical interventions^5^, which together may put deprived populations at greater risk of contracting COVID-19. Due to the lower incidence and mortality of COVID-19 in China, it is difficult to analyze how social inequalities may have impact COVID-19 infections and related health outcomes in China. However, our analysis provides some evidence to support that there exist evident social inequalities in information-seeking reactions to and awareness of COVID-19 in China, potentially exacerbating existing inequalities in COVID-19 related physical and mental health comes for both short and long term^13,34^.

Our study is subject to several limitations. First, our study only attempts to use the analysis of internet users' information-seeking behavior to reflect public concern about COVID-19. Although Baidu search is the most commonly used search engine in China with the highest market share, our findings could not be generalized to people that do not have access to the Internet. Second, as a disproportionately higher fraction of individuals without access to the internet have low SES and lower level of health literacy^35^, we may have underestimated the inequalities in the information-seeking response among regions with different SES. Third, due to the lack of data, we were not able to examine the influence of mass media, which likely mediated internet searches, although the reverse is also possible (that is, internet searches could also mediate mass media exposure)^36,37^. Lastly, we were not able to explore how individuals reacted to a health crisis using more disaggregated, individual-level data, such as data from surveys. We were able to examine how patterns of information-seeking responses differed according to the area-level HDI metric and used this measure to generate a hypothesis about potential associations with respect to individual factors, including education and income.

We used Baidu search data to analyze the first wave of the COVID-19 epidemic in China and several subsequent small outbreaks and found that there was an unprecedented increase in public awareness of the COVID-19 epidemic in China, and that the several subsequent outbreaks also sparked intense concern among internet users across China. Changes in the patterns of search interest in COVID-19 in each province of China were nearly synchronous during the first wave of the COVID-19 pandemic and subsequent local outbreaks, irrespective of the location of the epicenter of each outbreak and the variation in pandemic severity across the country. However, social inequalities in public response and awareness of COVID-19 were apparent, with less search interest observed in less developed areas compared with developed areas.

## Materials and methods

### Data

Baidu is the most popular search engine in China. The Baidu index (BI) is measured as the weighted frequency of unique searches for a search keyword or phrase relative to total search volume on Baidu on a given day^38^. We used the Baidu index of the most commonly used COVID-19 related search terms ("新型冠状病毒[*Xin Xing Guan Zhuang Bing Du*: novel coronavirus]", "疫情[*Yi Qing*: epidemic])", "新型冠状病毒肺炎[*Xin Guan Bing Du*: novel coronavirus pneumonia ]", "肺炎[*Fei Yan*: pneumonia]", "新冠病毒[*Xin Guan Bing Du*: *Xi Guan* virus]", "新冠肺炎[*Xin Guan Fei Yan:* Xin Guan pneumonia] ", "covid", "covid-19", "ncov", "2019-ncov", "NCP[novel coronavirus pneumonia]", and "coronavirus") for 31 provinces from January 1 2017 to March 15 2021 in China to reflect public interest in COVID-19 during the pandemic in China. The provincial daily confirmed COVID-19 cases were retrieved from official daily report^39^. The provincial-level human development index (HDI), an area-level measure of socioeconomic status, was retrieved from the China National Human Development Report 2019 to reflect regional-level SES^11,40^. A key advantage to examining an area-level measure in this context is its utility in providing evidence to help guide community-level interventions and policies. Other area-level measures by province, including the Gross National Product per person (GNPPP), the average number of years of education received by people ages 25 and older, and life expectancy at birth, which were used to calculate the HDI index, were extracted from the statistical yearbook and publicly available reports.

Our aim was to examine a series of three interrelated research questions, including (1) Did the Covid-19 outbreak lead to statistically significant increase in the Baidu Index of Covid-19 related terms? (2) What was the magnitude of the increases in searches compared to pre-Covid-19 forecasted trends, and how did these increases differ by regions with different social-economic development levels, and (3) Did the collective attention diminish toward pre-Covid-19 levels after the pandemic apex, and how did this differ according to the human development index (HDI)?

### Ethical statements

This study was exempt from institutional review oversight since the data are publicly accessible and aggregated at population level. Methods were carried out in accordance with relevant guidelines and regulations.

### Statistical analysis

After the initial exploration of search indices over time, we adopted an interrupted time series design to examine the effects of Covid-19. The effect was modeled using a segmented log-normal regression parameterization^41–44^ defining both pre-Covid trends (January 1 2017–December 30 2019), and distinct post-Covid periods that reflected different pandemic periods as experienced within China. Due to known large provincial-level heterogeneity in baseline levels as well as long-term trends, we employed mixed-effects models with random intercepts and random slopes over time, with individual provinces representing the random effects^42^. To adjust for observed seasonal and weekly cyclical patterns, we included fixed-effects of monthly and weekly indicator variables in all models. The Poisson model equation estimating the daily search index was expressed as follows:

$$E\left(ln \left({Index}_{ it}\right) \right) ={\beta }_{0i}+{\beta }_{1i} T+{\beta }_{c} Covid*{{HDI}_{i}}+{\beta }_{c1} Covid1*{{HDI}_{i}}+{\beta }_{cs1} {T}_{1}*{{HDI}_{i}}+{\beta }_{c2} Covid2*{{HDI}_{i}}+{\beta }_{cs2} Covid2*{{HDI}_{i}}+{\beta }_{c3} Covid3*{{HDI}_{i}}+{\beta }_{cs3} {T}_{3}*{{HDI}_{i}}+{\beta }_{c4} Covid4*{{HDI}_{i}}+{\beta }_{cs4} {T}_{4}*{{HDI}_{i}}+{\sum }_{m=2}^{12} {\beta }_{ Mm} Month+ {\sum }_{d=2}^{7} {\beta }_{ Dd}Day$$

In the model, ***Index***_***it***_ denotes the value of the search index in province *i* at time *t*. ***HDI***_***i***_ is the HDI category (low, middle or high) for province *i*. ***β***_***0i***_ represents the model intercept with both a fixed effect and province-level random effects, ***β***_***1i***_ represents the underlying pre-Covid-19 secular trends with both a fixed effect and province-level random effects. The five distinct indicator variables (***Covid***,*** Covid1***,*** Covid2***,*** Covid3*** and*** Covid4***) are used to define the exposures or intervals: 1) December 31 2019, the estimated start of the first Covid-19 wave; 2) 18 January 2020 (right before the official announcement of human-to-human transmission via mass media) to Jan 25 January 2020 (shortly after the lockdown and the estimated peak of daily search index in the initial Covid-19 wave); 3) a second outbreak in Beijing starting on June 11; 4) the outbreak in Qingdao starting on October 12 2020; 5) the outbreak in Shijiazhuang starting on January 3 2021. ***T*** is the time (days) elapsed since the start of the study, and ***T***_***1***_, ***T***_***2***_, ***T***_***3***_, and ***T***_***4***_ represent the days since the estimated peak (25 January 2020, June 17 2020, October 12 2020 and January 7 2021) of the daily search index associated with each distinct exposure, respectively. We interacted the main effect terms with strata of HDI categories, examining the extent to which the change in search index associated with each exposure differed by area-level socioeconomic status. ***Month*** and ***Day*** are individual dummy variables indexing month of the year using the month of January as the reference category, and the day of the week using Friday as the reference category respectively. An AR (1) correlation structure was used to accommodate autocorrelation in residual errors. In order to estimate the association of each component of HDI with the change of search index, we replaced ***HDI***_***i***_ in the equation by standardized HDI (continuous variable), GNPPP, years of education or life expectancy and repeated all the analyses.

We employed a linear mixed-model with logarithmic transformation of the independent variable and a normal residual distribution^45^. We used a mixed-effects log-normal model rather than a negative bionomial or Poisson model for three reasons. First, an attempt to run these generalized linear models with log link (e.g. Poisson and negative binomial model) failed to converge without simplifications such as the elimination of an AR(1) correlation structure in residual errors and the elimination of provincial-level random slopes. Second, mixed effect log-normal models provided a better fit to the data patterns than the fixed-effect log-normal models and generalized linear models judged by Akaike’s Information Criteria (AIC) and Bayesian Information Criteria (BIC). Third, there was no evidence of issues with heteroscedasticity in the residuals departure from a normal distribution in the error distribution when using the log normal model.

All analyses were conducted in R-version-4.0.2 using data obtained March 31, 2021. A two-sided alpha value of 0.05 indicated statistical significance. In order to maintain a family-wise alpha (type I error rate) at 0.05 over multiple comparisons, the Bonferroni correction was employed for predefined exposures for each of the 3 HDI categories. This defined a test-specific significance level of 0.05/(number of tests in analysis-rank of p value from lowest to highest + 1)^46^. This study is reported as per the Strengthening the Reporting of Observational Studies in Epidemiology (STROBE) guidelines for cohort studies.

## Supplementary Information

Supplementary Information 1.

Supplementary Information 2.

## Data Availability

The datasets used and/or analysed during the current study available from the corresponding authors on reasonable request.

## Abbreviations

HDI: Human development index
GNP: Gross national product
IQR: Interquartile range
HHT: Human-to-human transmission
NPIs: Non-pharmaceutical interventions
