# Supplementary material for: Geographic social inequalities in information-seeking response to the COVID-19 pandemic in China: longitudinal analysis of Baidu Index
Source: Sci Rep. 2022 Jul 18;12:12243. doi: 10.1038/s41598-022-16133-2 (PMC9293890; doi:10.1038/s41598-022-16133-2)
Supplement: Supplementary file 1 — Supplementary Information 1. [file 41598_2022_16133_MOESM1_ESM.pdf]

Supplementary Materials

Table S1 Univariate analysis of the association between SES factors and change in search index

	**HDI**		**GNPPP**		**Education**		**Life Expectancy**	
	Ratio of RR (95% CI)	p-value	Ratio of RR (95% CI)	p-value	Ratio of RR (95% CI)	p-value	Ratio of RR (95% CI)	p-value
**Pre-pandemic**								
Yearly change Jan 1 2016 - Dec 30 2019	1.01 (0.99, 1.02)	.2929	1.00 (0.99, 1.02)	.7306	1.01 (0.99, 1.	.3665	1.01 (1.00, 1.03)	.1459
**Initial COVID-19 Wave**								
Level Change on Dec 31 2019	1.09 (1.05, 1.12)	<.0001	1.06 (1.02, 1.09)	.0009	1.09 (1.06, 1.13)	<.0001	1.07 (1.04, 1.11)	<.0001
Level change Jan 18 (HHT announced) - Jan 25 2020 (lockdown)	1.04 (1.01, 1.08)	.0395	0.96 (0.92, 0.99)	.0232	1.06 (1.02, 1.10)	.0015	1.06 (1.02, 1.10)	.0015
Weekly change Jan 25 - Jun 10 2020	1.00 (0.99, 1.00)	.0012	1.00 (1.00, 1.00)	.2014	1.00 (0.99, 1.00)	.0002	1.00 (0.99, 1.00)	.0003
**Beijing Outbreak**								
Level change Jun 11- Jun 17 2020	1.06 (1.02, 1.10)	.0009	1.05 (1.01, 1.09)	.0052	1.07 (1.03, 1.10)	.0002	1.05 (1.01, 1.08)	.0085
Weekly change Jun 17 - Oct 11 2020	0.99 (0.99, 0.99)	<.0001	0.99 (0.99, 1.00)	<.0001	0.99 (0.99, 0.99)	<.0001	0.99 (0.99, 0.99)	<.0001
**Qingdao Outbreak**								
Level Change on Oct 12th	1.04 (1.00, 1.08)	.0324	1.05 (1.01, 1.09)	.0112	1.04 (1.00, 1.08)	.0632	1.03 (0.99, 1.07)	.1577
Weekly change in winter wave Oct 12 2020 - Jan 3 2021	1.00 (0.99, 1.00)	.7459	1.00 (0.99, 1.00)	.0445	1.00 (1.00, 1.01)	.6399	1.00 (0.99, 1.01)	.3456
**Shijiazhuang Outbreak**								
Level change Jan 3- Jan 7 2021	1.11 (1.06, 1.16)	<.0001	1.05 (1.00, 1.10)	.0362	1.11 (1.06, 1.16)	<.0001	1.12 (1.07, 1.17)	<.0001
Weekly change Jan 7- Mar 15 2021	0.98 (0.97, 0.99)	<.0001	0.99 (0.98, 1.00)	.0023	0.98 (0.94, 0.99)	<.0001	0.98 (0.97, 0.98)	<.0001

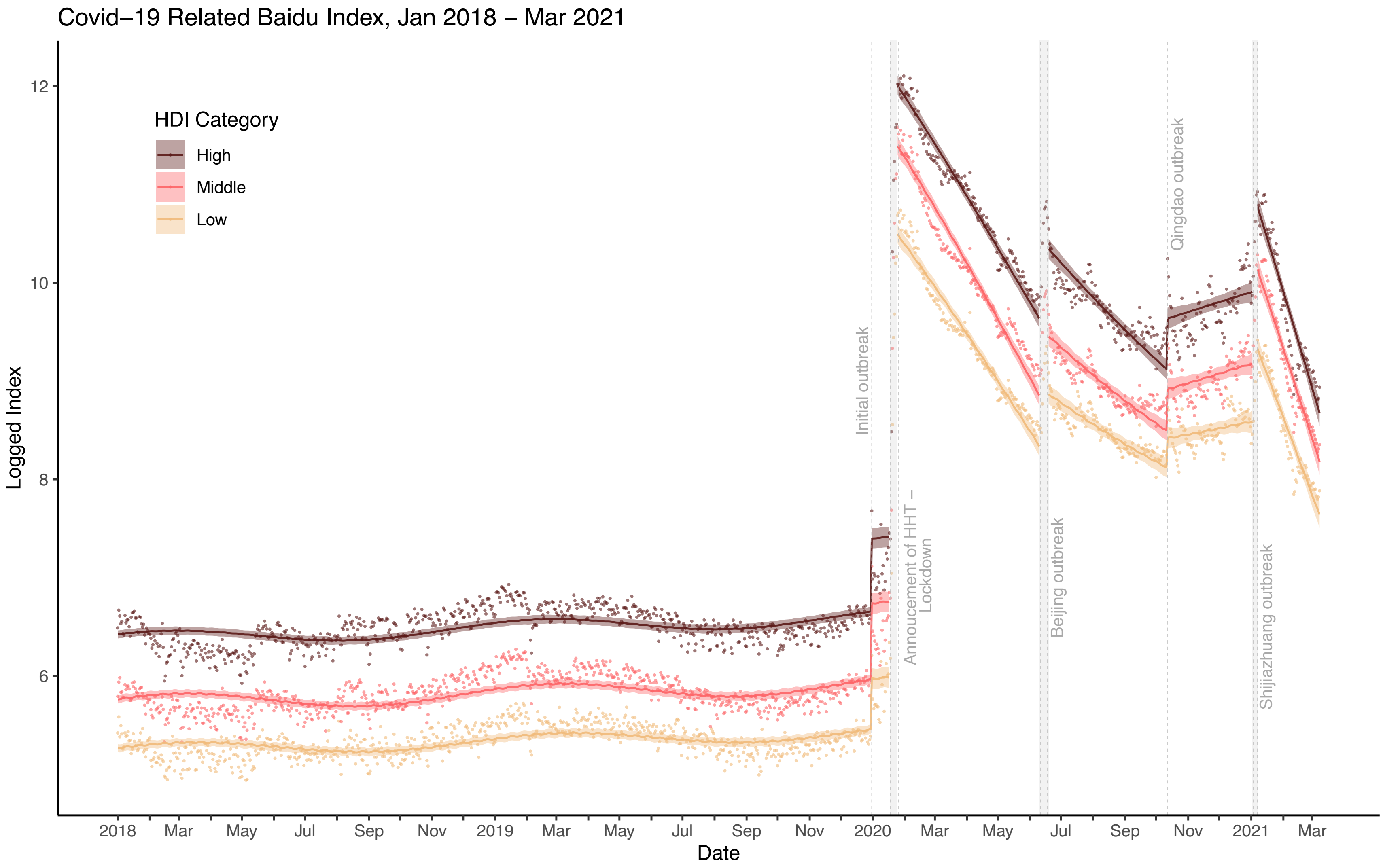

Figure S1. Baidu search index by regional HDI over time.
