# Supplementary material for: Geographic social inequalities in information-seeking response to the COVID-19 pandemic in China: longitudinal analysis of Baidu Index
Source: Sci Rep. 2022 Jul 18;12:12243. doi: 10.1038/s41598-022-16133-2 (PMC9293890; doi:10.1038/s41598-022-16133-2)
Supplement: Supplementary file 2 — Supplementary Information 2. [file 41598_2022_16133_MOESM2_ESM.docx]

STROBE Statement—checklist of items that should be included in reports of observational studies

|  | Item No. | Recommendation | Page  No. | Relevant text from manuscript |
| --- | --- | --- | --- | --- |
| **Title and abstract** | 1 | (*a*) Indicate the study’s design with a commonly used term in the title or the abstract | 2 | We used an interrupted time-series analysis to examine the change in Baidu Search Index of selected COVID-19 related terms associated with the COVID-19 derived exposure variables. |
|  |  | (*b*) Provide in the abstract an informative and balanced summary of what was done and what was found | 2 | In this longitudinal study of nearly one billion internet users, we found synchronous increases in COVID-19 related searches during the first wave of the COVID-19 pandemic and subsequent local outbreaks, irrespective of the location and severity of each outbreak. The most precipitous increase occurred in the week when most provinces activated their highest level of response to public health emergencies. Search interests increased more as Human Development Index (HDI) -an area level measure of socioeconomic status – increased. Searches on the index began to decline nationwide after the initiation of mass-scale lockdowns, but statistically significant increases continued to occur in conjunction with the report of major sporadic local outbreaks. The intense interest in COVID-19 related information at virtually the same time across different provinces indicates that the Chinese government utilizes multiple channels to keep the public informed of the pandemic. Regional socioeconomic status influenced search patterns. |
| Introduction | | | |  |
| Background/rationale | 2 | Explain the scientific background and rationale for the investigation being reported | 3 | Previous survey-based studies have shown that individuals with lower socioeconomic status have lower awareness, concern and knowledge about certain infectious disease during the outbreaks^7-10^. Therefore, collective public information-seeking behaviors may vary across the 31 provinces in China in relation to differences in socioeconomic development ^11^. However, few studies about inequities in awareness or response to COVID-19 in China have been conducted to date, in part due to the failure to capture the social determinants in the health information system in China. |
| Objectives | 3 | State specific objectives, including any prespecified hypotheses | 4, 10 | The investigation of the intensity of internet search interests can therefore be used to examine potential differences in patterns of information-seeking reactions (and by extension, health literacy and health outcomes) regarding the pandemic by levels of socioeconomic status.  Our aim was to examine a series of three interrelated research questions, including (1) Did the Covid-19 outbreak lead to statistically significant increase in the Baidu Index of Covid-19 related terms? (2) What was the magnitude of the increases in searches compared to pre-Covid-19 forecasted trends, and how did these increases differ by regions with different social-economic development levels, and (3) Did the collective attention diminish toward pre-Covid-19 levels after the pandemic apex, and how did this differ according to the human development index (HDI)? |
| Methods | | | |  |
| Study design | 4 | Present key elements of study design early in the paper | 11 | After the initial exploration of search indices over time, we adopted an interrupted time series design to examine the effects of Covid-19. |
| Setting | 5 | Describe the setting, locations, and relevant dates, including periods of recruitment, exposure, follow-up, and data collection | 10 | We used the Baidu index of the most commonly used COVID-19 related search terms ("新型冠状病毒[*Xin Xing Guan Zhuang Bing Du*: novel coronavirus]", "疫情[*Yi Qing*: epidemic])", "新型冠状病毒肺炎[*Xin Guan Bing Du*: novel coronavirus pneumonia ]", "肺炎[*Fei Yan*: pneumonia]", "新冠病毒[*Xin Guan Bing Du*: *Xi Guan* virus]", "新冠肺炎[*Xin Guan Fei Yan:* Xin Guan pneumonia] ", "covid", "covid-19", "ncov", "2019-ncov", "NCP[novel coronavirus pneumonia]", and "coronavirus") for 31 provinces from January 1 2017 to March 15 2021 in China to reflect public interest in COVID-19 during the pandemic in China. |
| Participants | 6 | (*a*) *Cohort study*—Give the eligibility criteria, and the sources and methods of selection of participants. Describe methods of follow-up  *Case-control study*—Give the eligibility criteria, and the sources and methods of case ascertainment and control selection. Give the rationale for the choice of cases and controls  *Cross-sectional study*—Give the eligibility criteria, and the sources and methods of selection of participants | 10 | We used the Baidu index of the most commonly used COVID-19 related search terms ("新型冠状病毒[*Xin Xing Guan Zhuang Bing Du*: novel coronavirus]", "疫情[*Yi Qing*: epidemic])", "新型冠状病毒肺炎[*Xin Guan Bing Du*: novel coronavirus pneumonia ]", "肺炎[*Fei Yan*: pneumonia]", "新冠病毒[*Xin Guan Bing Du*: *Xi Guan* virus]", "新冠肺炎[*Xin Guan Fei Yan:* Xin Guan pneumonia] ", "covid", "covid-19", "ncov", "2019-ncov", "NCP[novel coronavirus pneumonia]", and "coronavirus") for 31 provinces from January 1 2017 to March 15 2021 in China to reflect public interest in COVID-19 during the pandemic in China. |
|  |  | (*b*) *Cohort study*—For matched studies, give matching criteria and number of exposed and unexposed  *Case-control study*—For matched studies, give matching criteria and the number of controls per case |  |  |
| Variables | 7 | Clearly define all outcomes, exposures, predictors, potential confounders, and effect modifiers. Give diagnostic criteria, if applicable | 10, 11 | The provincial-level human development index (HDI), an area-level measure of socioeconomic status, was retrieved from the China National Human Development Report 2019 to reflect regional-level SES ^11, 40^. A key advantage to examining an area-level measure in this context is its utility in providing evidence to help guide community-level interventions and policies. Other area-level measures by province, including the Gross National Product per person (GNPPP), the average number of years of education received by people ages 25 and older, and mean life expectancy at birth, which were used to calculate the HDI index, were extracted from the statistical yearbook and publicly available reports.  The five distinct indicator variables (***Covid, Covid1, Covid2, Covid3 and Covid4***) are used to define the exposures or intervals: 1) December 31 2019, the estimated start of the first Covid-19 wave; 2) 18 January 2020 (official announcement of human-to-human transmission) to Jan 25 January 2020 (shortly after the lockdown and the estimated peak of daily search index in the initial Covid-19 wave); 3) a second outbreak in Beijing starting on June 11; 4) the outbreak in Qingdao starting on October 12 2020; 5) the outbreak in Shijiazhuang starting on January 3 2021. ***T*** is the time (days) elapsed since the start of the study, and ***T_1_, T_2_, T_3_,*** and ***T_4_*** represent the days since the estimated peak (25 January 2020, June 17 2020, October 12 2020 and January 7 2021) of the daily search index associated with each distinct exposure, respectively. |
| Data sources/ measurement | 8* | For each variable of interest, give sources of data and details of methods of assessment (measurement). Describe comparability of assessment methods if there is more than one group | *10, 11* |  |
| Bias | 9 | Describe any efforts to address potential sources of bias | 12 | ***Month*** and ***Day*** are individual dummy variables indexing month of the year using the month of January as the reference category, and the day of the week using Friday as the reference category respectively. |
| Study size | 10 | Explain how the study size was arrived at |  |  |

Continued on next page

| Quantitative variables | 11 | Explain how quantitative variables were handled in the analyses. If applicable, describe which groupings were chosen and why | 10 | The provincial-level human development index (HDI), an area-level measure of socioeconomic status, was retrieved from the China National Human Development Report 2019 to reflect regional-level SES |
| --- | --- | --- | --- | --- |
| Statistical methods | 12 | (*a*) Describe all statistical methods, including those used to control for confounding | 11 | The effect was modeled using a segmented log-normal regression parameterization^27^ defining both pre-Covid trends (January 1 2017 – December 30 2019), and distinct post-Covid periods that reflected different pandemic periods as experienced within China. |
|  |  | (*b*) Describe any methods used to examine subgroups and interactions | 11 | We interacted the main effect terms with strata of HDI categories, examining the extent to which the change in search index associated with each exposure differed by area-level socioeconomic status. |
|  |  | (*c*) Explain how missing data were addressed | NA | No missing data |
|  |  | (*d*) *Cohort study*—If applicable, explain how loss to follow-up was addressed  *Case-control study*—If applicable, explain how matching of cases and controls was addressed  *Cross-sectional study*—If applicable, describe analytical methods taking account of sampling strategy |  |  |
|  |  | (*e*) Describe any sensitivity analyses |  |  |
| Results | | | | |
| Participants | 13* | (a) Report numbers of individuals at each stage of study—eg numbers potentially eligible, examined for eligibility, confirmed eligible, included in the study, completing follow-up, and analysed | Not applicable |  |
|  |  | (b) Give reasons for non-participation at each stage | Not applicable |  |
|  |  | (c) Consider use of a flow diagram | Not applicable |  |
| Descriptive data | 14* | (a) Give characteristics of study participants (eg demographic, clinical, social) and information on exposures and potential confounders | Not applicable |  |
|  |  | (b) Indicate number of participants with missing data for each variable of interest | Not applicable |  |
|  |  | (c) *Cohort study*—Summarise follow-up time (eg, average and total amount) | Not applicable |  |
| Outcome data | 15* | *Cohort study*—Report numbers of outcome events or summary measures over time | *4* | Across the 31 provinces and regions in mainland China, the mean daily search index at the national level for Covid-19 related terms was 4, 533 (IQR (Interquartile Range) =1, 301) before the outbreak (January 1 2017 to December 30 2019), and 314, 718 (IQR=445, 074) after the outbreak (December 31 2019 to March 15 2021). |
|  |  | *Case-control study—*Report numbers in each exposure category, or summary measures of exposure |  |  |
|  |  | *Cross-sectional study—*Report numbers of outcome events or summary measures |  |  |
| Main results | 16 | (*a*) Give unadjusted estimates and, if applicable, confounder-adjusted estimates and their precision (eg, 95% confidence interval). Make clear which confounders were adjusted for and why they were included | 4, 5 |  |
|  |  | (*b*) Report category boundaries when continuous variables were categorized |  |  |
|  |  | (*c*) If relevant, consider translating estimates of relative risk into absolute risk for a meaningful time period |  |  |

Continued on next page

| Other analyses | 17 | Report other analyses done—eg analyses of subgroups and interactions, and sensitivity analyses | 7 | For each exposure, the difference associated with GNPPP, education year or life expectancy in the directions and magnitudes of both immediate and gradual effect across provinces was similar to the difference associated with HDI. |
| --- | --- | --- | --- | --- |
| Discussion | | | | |
| Key results | 18 | Summarise key results with reference to study objectives | 7 | Our study found that, in January 2020, the outbreak of the Wuhan epidemic triggered an increase in search terms for COVID-19 among Internet users in different regions. In particular, this increasing trend was most sharply observed between 18 January and 25 January 2020, a period when Chinese television, radio and newspapers reported the confirmation of human-to-human transmission of SARS-CoV-2, greatly increasing public awareness of the threat of the disease. |
| Limitations | 19 | Discuss limitations of the study, taking into account sources of potential bias or imprecision. Discuss both direction and magnitude of any potential bias | 9 | Our study is subject to several limitations. First, our study only attempts to use the analysis of internet users' information-seeking behavior to reflect public concern about COVID-19. Although Baidu search is the most commonly used search engine in China with the highest market share, our findings could not be generalized to people that do not have access to the Internet.  Second, as a disproportionately higher fraction of individuals without access to the internet have low SES and lower level of health literacy,^35^ we may have underestimated the inequalities in the information-seeking response among regions with different SES’s. Third, due to the lack of data, we were not able to examine the influence of mass media, which likely mediated internet searches, although the reverse is also possible (that is, internet searches could also mediate mass media exposure).^36, 37^.Lastly, we were not able to explore how individuals reacted to a health crisis using more disaggregated, individual-level data, such as data from surveys. We were able to examine how patterns of information-seeking responses differed according to the area-level HDI metric and used this measure to generate a hypothesis about potential associations with respect to individual factors, including education and income. |
| Interpretation | 20 | Give a cautious overall interpretation of results considering objectives, limitations, multiplicity of analyses, results from similar studies, and other relevant evidence | 9 | We used Baidu search data to analyze the first wave of the COVID-19 epidemic in China and several subsequent small outbreaks and found that there was an unprecedented increase in public awareness of the COVID-19 epidemic in China, and that the several subsequent outbreaks also sparked intense concern among internet users across China. Changes in the patterns of search interest in COVID-19 in each province of China were nearly synchronous during the first wave of the COVID-19 pandemic and subsequent local outbreaks, irrespective of the location of the epicenter of each outbreak and the variation in pandemic severity across the country. However, social inequalities in public response and awareness of COVID-19 were apparent, with less search interest observed in less developed areas compared with developed areas. |
| Generalisability | 21 | Discuss the generalisability (external validity) of the study results |  |  |
| Other information | |  | | |
| Funding | 22 | Give the source of funding and the role of the funders for the present study and, if applicable, for the original study on which the present article is based | 9 | National Natural Science Foundation of China (72074130)  Spring Breeze Foundation of Tsinghua University (20203080035).  AIR@InnoHK administered by Innovation and Technology Commission. |

*Give information separately for cases and controls in case-control studies and, if applicable, for exposed and unexposed groups in cohort and cross-sectional studies.

**Note:** An Explanation and Elaboration article discusses each checklist item and gives methodological background and published examples of transparent reporting. The STROBE checklist is best used in conjunction with this article (freely available on the Web sites of PLoS Medicine at http://www.plosmedicine.org/, Annals of Internal Medicine at http://www.annals.org/, and Epidemiology at http://www.epidem.com/). Information on the STROBE Initiative is available at www.strobe-statement.org.
